# Expression of apoptosis repressor with caspase recruitment domain (ARC) in familial adenomatous polyposis (FAP) adenomas and its correlation with DNA mismatch repair proteins, p53, Bcl-2, COX-2 and beta-catenin

**DOI:** 10.1186/s12964-020-00702-x

**Published:** 2021-02-12

**Authors:** Christoph Roser, Csaba Tóth, Marcus Renner, Esther Herpel, Peter Schirmacher

**Affiliations:** 1 Institute of Pathology, Heidelberg University Hospital, Im Neuenheimer Feld 224, 69120 Heidelberg, Germany; 2 Department of Orthodontics and Dentofacial Orthopaedics, Heidelberg University Hospital, Im Neuenheimer Feld 400, 69120 Heidelberg, Germany; 3 Tissue Bank of the National Center for Tumor Diseases (NCT), Im Neuenheimer Feld 224, 69120 Heidelberg, Germany; 4 Trier MVZ for Histology, Cytology and Molecular Diagnostics, Max-Planck-Straße 5, 54296 Trier, Germany

**Keywords:** ARC, Apoptosis, FAP, Bcl-2, COX-2, p53, β-catenin, Mismatch repair protein

## Abstract

**Background:**

Colorectal familial adenomatous polyposis (FAP) adenomas exhibit a uniform pathogenetic basis caused by a germline mutation in the adenomatous polyposis gene (*APC*), but the molecular changes leading to their development are incompletely understood. However, dysregulated apoptosis is known to substantially affect the development of colonic adenomas. One of the key regulatory proteins involved in apoptosis is apoptosis repressor with caspase recruitment domain (ARC).

**Methods:**

The expression of nuclear and cytoplasmic ARC in 212 adenomas from 80 patients was analyzed by immunohistochemistry. We also compared expression levels of ARC with the expression levels of p53, Bcl-2, COX-2, and MMR proteins. Statistical analyses were performed by Spearman’s rank correlation and linear regression test.

**Results:**

ARC was overexpressed in the nuclei and cytoplasm of most FAP adenomas investigated. Cytoplasmic ARC staining was moderately stronger (score 2) in 49.1% (n = 104/212) and substantially stronger (score 3) in 32.5% (n = 69/212) of adenomas compared to non-tumorous colorectal mucosa. In 18.4% (n = 39/212) of adenomas, cytoplasmic ARC staining was equivalent to that in non-tumorous mucosa.

Nuclear expression of ARC in over 75% of cells was present in 30.7% (n = 65/212) of investigated adenomas, and nuclear expression in 10–75% of cells was detected in 62.7% (n = 133/212). ARC expression in under 10% of nuclei was found in 6.6% (n = 14/212) of adenomas.

The correlation between nuclear ARC expression and cytoplasmic ARC expression was highly significant (*p* = 0.001). Moreover, nuclear ARC expression correlated positively with overexpression of Bcl-2, COX-2 p53 and β-catenin. Cytoplasmic ARC also correlated with overexpression of Bcl-2. Sporadic MMR deficiency was detected in very few FAP adenomas and showed no correlation with nuclear or cytoplasmic ARC.

**Conclusions:**

Our results demonstrated that both cytoplasmic and nuclear ARC are overexpressed in FAP adenomas, thus in a homogenous collective. The highly significant correlation between nuclear ARC and nuclear β-catenin suggested that ARC might be regulated by β-catenin in FAP adenomas. Because of its further correlations with p53, Bcl-2, and COX-2, nuclear ARC might play a substantial role not only in carcinomas but also in precursor lesions.

**Video Abstract**

## Background

Familial adenomatous polyposis (FAP) is an autosomal dominant disease with a prevalence of 3–10 people per 100 000. It is characterized by the development of up to several thousand adenomas in the rectum and colon in the second decade of life [1, 2]. It is usually caused by a mutation in the adenomatous polyposis coli (*APC*) gene, which is located on chromosome 5q21-q22. The *APC* gene codes for the APC protein, which participates in the degradation of the proto-oncoprotein β-catenin [3–5]. Approximately 1% of all colorectal carcinomas are caused by FAP [2].

Dysregulated apoptosis is known to strongly affect the development of colonic adenomas. Apoptosis repressor with caspase recruitment domain (ARC) is an inhibitor of apoptosis and affects both the intrinsic and extrinsic apoptosis pathways [6–8].

The ARC protein is encoded by the *NOL3* gene, which is located on the long arm of human chromosome 16q21-23. It consists of an N-terminal caspase recruitment domain (CARD) and a C-terminal region that harbors many proline-glutamate (P/E) tandem repeats. The CARD belongs to the death domain superfamily, and its amino-acid structure resembles that of caspases 2 and 9 and the apoptotic protease-activating factor 1 (Apaf1) [7, 9, 10].

ARC interacts selectively with caspases 2 and 8 but not with caspases 1, 3, or 9. It inhibits the enzyme activity of caspase 8 and thus impedes apoptosis [7, 8]. Moreover, its direct interaction with the death receptor FAS and the adaptor protein Fas-associated death domain (FADD) prevents the assembly of the death-inducing signaling complex (DISC), which is essential for the initiation of extrinsic apoptosis [11]. But the ARC-mediated effect not only operates through the inhibition of caspases. ARC has been shown to interact with the pro-apoptotic protein Bax, which prevents its activation and the subsequent mitochondrial release of cytochrome c [6, 12]. In addition, ARC interacts with the Bcl-2 homology (BH)3-only-proteins Bad and Puma, which normally activate Bax and Bak and deactivate anti-apoptotic Bcl-2 proteins [6, 11]. ARC is also capable of binding large amounts of Ca^2+^ to its P/E domain, thereby reducing the apoptotic potential of a cell by blocking endoplasmic mitochondrial Ca^2+^ transfer [8].

ARC transcription is mainly regulated via the Ras/Raf/MAPK and Wnt/β-catenin signaling pathways. In breast and colon cancer cell lines, Ras is shown to activate the ARC promoter Nol3 in a MEK/ERK-dependent matter. Moreover, Ras is capable to increase ARC on mRNA and protein levels in normal mamillary epithelium, hyperplastic regions of the mamillary epithelium and in derived tumours, while knockdown of Ras was shown to decrease both levels in breast cancer [13]. In acute myeloid leukemia (AML) cells, Carter et al. evidenced transcriptional regulation of ARC via the Wnt/β-Catenin pathway. They showed ARC to be part of a positive ARC-IL1b/Cox-2/PGE2/β-Catenin feedback loop, which resulted in a β-Catenin mediated transcriptional regulation of ARC [14].

Post-translational ARC activity is mainly regulated by protein kinase CK2, which phosphorylates ARC at T149 [15, 16]. ARC expression is also controlled by polyubiquitination and subsequent degradation governed by the p53-induced ubiquitin E3 ligase, MDM2. In addition, ARC down-regulation is a known consequence of p53 activation; this indicates that p53 is able to transcriptionally target ARC [17, 18]. In contrast, Ras prevents post-translational degradation of ARC by modifying it to protect it from polyubiquitination and degradation [13].

Physiologically, ARC is expressed in the nucleus and cytoplasm of differentiated cardiac and skeletal muscle cells, neurons, and pancreatic beta cells [7, 19, 20]. However, ARC is also expressed in many different types of tumor, such as breast, lung, prostate, ovary, cervix, kidney, and colon, where it inhibits apoptosis in particular [21–26]. In addition to affecting tumor progression and the invasion of these tumors, it also affects the formation of metastases by increasing the number of circulating cancer cells [26]. Moreover, ARC contributes to chemo- and radiotherapy resistance by protecting tumor cells from apoptosis induced by chemotherapeutic agents, such as doxorubicin and cisplatin, or by gamma radiation [12, 27, 28].

Cytoplasmic ARC interacts with the apoptotic regulator proteins Bax, Bad, Puma, and procaspase 2, and with FAS and FADD, and its overexpression leads to cell immortalization [11, 21–24, 27, 29]. The function of nuclear ARC is less clear, but a nuclear shift in carcinoma cells [25] and nuclear ARC in dysplasia lesions have been described [21, 27].

Here, we show for the first time that ARC is overexpressed in the nucleus and cytoplasm of FAP adenoma cells, and that the correlation between its expression in both locations is highly significant. Furthermore, we prove that ARC expression correlates with the expression of p53, COX-2, and Bcl-2, which were previously shown to be involved in the development and progression of FAP adenomas [30–36]. We suggest that ARC might have a regulatory role in the development of FAP adenomas, and that nuclear ARC in particular might have a greater effect on carcinogenesis than previously thought.

## Methods

### Tissue samples

This study used formalin fixed paraffin-embedded (FFPE) surgical specimens from total proctocolectomies or colon resections carried out between 1992 and 2004. The specimens came from the archive of the Institute of Pathology at Heidelberg University Hospital. Adenomas from 80 patients (mean age 32.4 years; 57% men, 43% women) were included. From each patient, 1–11 adenomas were randomly selected for further analysis, resulting in 217 adenomas in total.

All tissue samples were provided by the tissue bank of the National Center for Tumor Diseases (NCT; Heidelberg, Germany) in compliance with the regulations of the tissue bank and the approval (approval nr.: 206/2005) of Heidelberg University’s ethics committee, in accordance with the ethical standards in the revised 1975 Declaration of Helsinki and its later revisions.

### Tissue microarray

A tissue microarrayer (AlphaMetrix; Roedermark, Germany) was used to create tissue microarray (TMA) blocks from the FFPE specimens. Three cores of adenoma tissue and one core of non-tumorous colorectal mucosa were punched from each specimen with a needle (diameter: 1.0 mm). Two skeletal muscle cores served as positive controls for ARC immunostaining.

### Immunohistochemistry

For IHC 4 µm thick tissue slides were used. These slices were deparaffinized using xylene in accordance with standard protocols and rehydrated with 95–96% ethanol, 70% ethanol, and distilled water. Before antibody incubation, all slices were pretreated with 3% hydrogen peroxide to block endogenous peroxidase. The following primary antibodies were used: ARC (H-150, 1:200, SCBT; Santa Cruz, California, USA), MLH1 (M1, ready-to-use [RTU], Ventana, Roche Diagnostics; Mannheim, Germany), MSH2 (G219-1129, RTU, Ventana, Roche Diagnostics), MSH6 (44, RTU, Ventana, Roche Diagnostics), p53 (DO-7, 1:100, Dako; Hamburg, Germany), Bcl-2 (SP66, RTU, Ventana, Roche Diagnostics), COX-2 (SP21, RTU, Ventana, Roche Diagnostics). Secondary antibody binding (all Dako, 1:200) was visualized by use of a streptavidin ABC kit (Dako), and nuclear counterstaining was performed by use of chromogen 3,3′-diaminobenzidine (Vector Laboratories; Peterborough, UK) and hematoxylin.

Primary antibodies were incubated at 4 °C overnight and then stained by use of the avidin–biotin complex peroxidase technique. Aminoethyl carbazole was used for visualization, and hematoxylin for nuclear counterstaining. All specimens were mounted in Aquatex (Merck; Darmstadt, Germany) under a glass cover slip.

The immunostained tissue microarray slices were scored and evaluated under a light microscope by two independent, blinded pathologists. In the case of disagreement, the slice in question was discussed by the evaluating pathologists until consensus was achieved. Stained slices were classified as non-evaluable if adenoma tissue was missing or if non-stained tissue was present on all of the respective TMA slices.

Staining for MSI proteins was evaluated according to the Bethesda guidelines [37], whereby score 1 = more than 10% of tumor-cell nuclei exhibit positive staining and score 0 = less than 10% of tumor-cell nuclei exhibit positive staining.

Immunostaining for p53 and nuclear β-catenin was evaluated on the basis of previously published scorings but without intensity scoring [23, 38], whereby score 0 = nuclear staining in less than 10% of tumor cells, score 1 = nuclear staining in up to 75% of tumor cells, and score 2 = nuclear staining in more than 75% of tumor cells.

The scoring system used to evaluate immunostaining for Bcl-2 was comparable to scales already used [39, 40]. In contrast to the scoring used by Tsuyama et al., further differentiation of expression was not conducted for specimens with staining in 10–75% of cells, and specimens showing expression in over 75% of tumor cells were evaluated as overexpressed.

A standardized evaluation method for COX-2 is not available [40]. In our study, the positivity of the cells was evaluated and was scored as follows. Score 0 = no expression or expression in less than 10% of tumor cells, score 1 = cytoplasmic expression in up to 75% of tumor cells, and score 2 = cytoplasmic expression in more than 75% of tumor cells.

To semi-quantitatively assess the intensity of ARC staining, we adapted a previously published scoring system to the TMA slices [21]. ARC expression levels in non-tumorous colon mucosa were used as the baseline for comparison (score 1). Cytoplasmic and nuclear ARC staining were scored separately.

For cytoplasmic ARC staining, the reference score (score 1) was the non-tumorous colorectal mucosa, which was also used as an internal positive control for immunohistochemistry. A four-stage scale was used for cytoplasmic ARC expression as follows. Score 0 = no staining of tumor cells or weaker staining than in the non-tumorous colon mucosa; score 1 = cytoplasmic staining equivalent to that in the non-tumorous colon mucosa, perceptible staining intensity at 4X; score 2 = moderate staining intensity, stronger than that in the non-tumorous colon mucosa; score 3 = diffuse and strong cytoplasmic staining, substantially stronger than that in the non-tumorous colon mucosa.

For nuclear ARC staining, a three-stage scale was used, with score 0 = no nuclear staining or staining in less than 10% of nuclei, score 1 = 10–75% of nuclei positive, and score 2 = more than 75% of nuclei positive.

### Statistical analysis

Statistical analyses were performed with SPSS statistics 25 (IBM; Endicott, New York, USA). Associations between ARC and COX-2, Bcl-2, p53, and MMR proteins were calculated by means of Spearman’s rank correlation and linear regression tests. Statistical significance was set at *p* < 0.05 and *p* < 0.01.

## Results

### ARC is overexpressed in the nuclei and cytoplasm of FAP adenomas

**Fig. 1:**
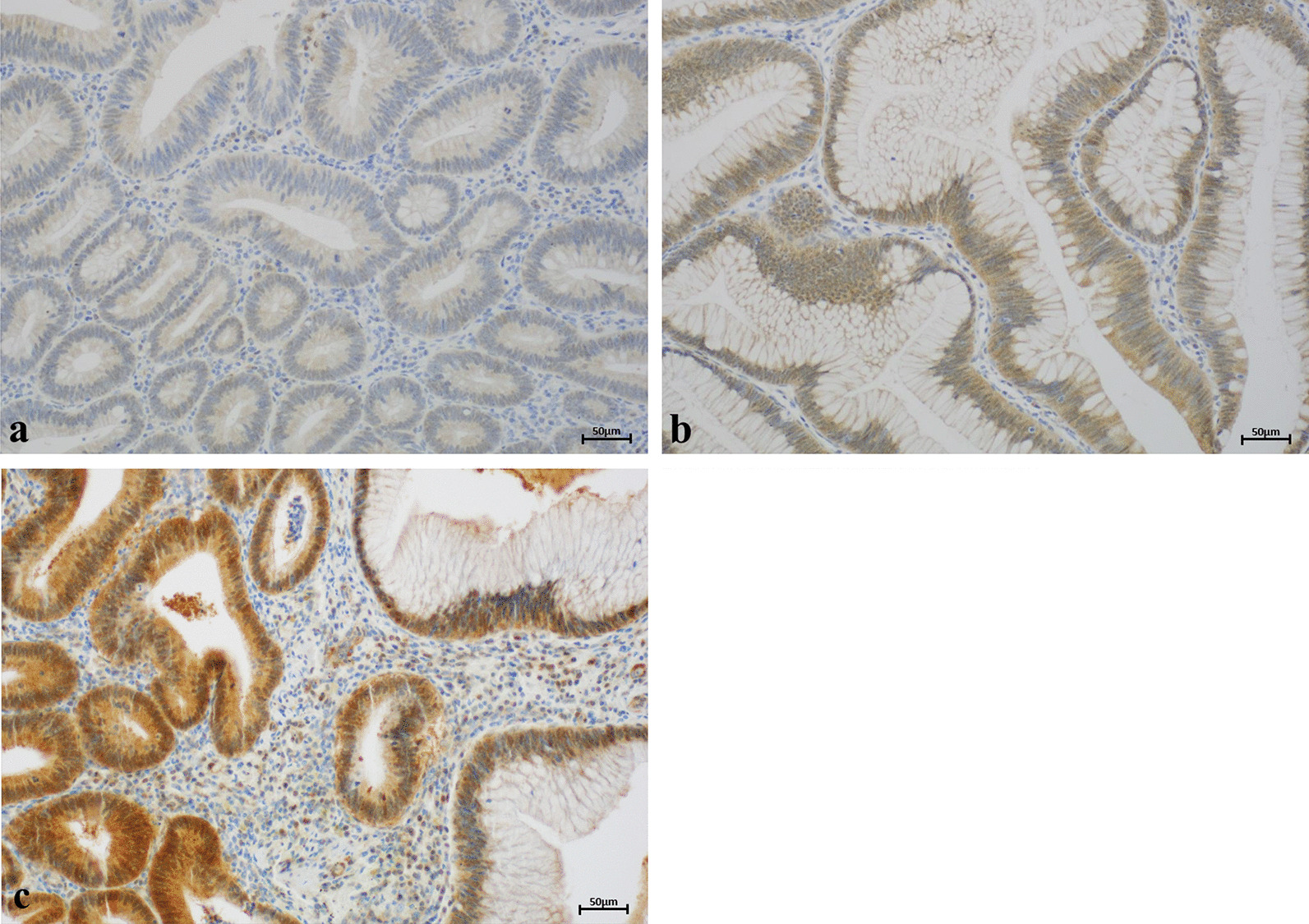
Representative results of cytoplasmic ARC staining. Only in 18.4% of FAP adenomas, cytoplasmic staining was equivalent to that in non-tumorous mucosa (score 1; **a**). In most of the examined FAP adenomas cytoplasmic ARC was overexpressed. Cytoplasmic overexpression was moderate (score 2; **b**) in 49.1% and strong (score 3; **c**) in 32.5% of adenomas

**Fig. 2:**
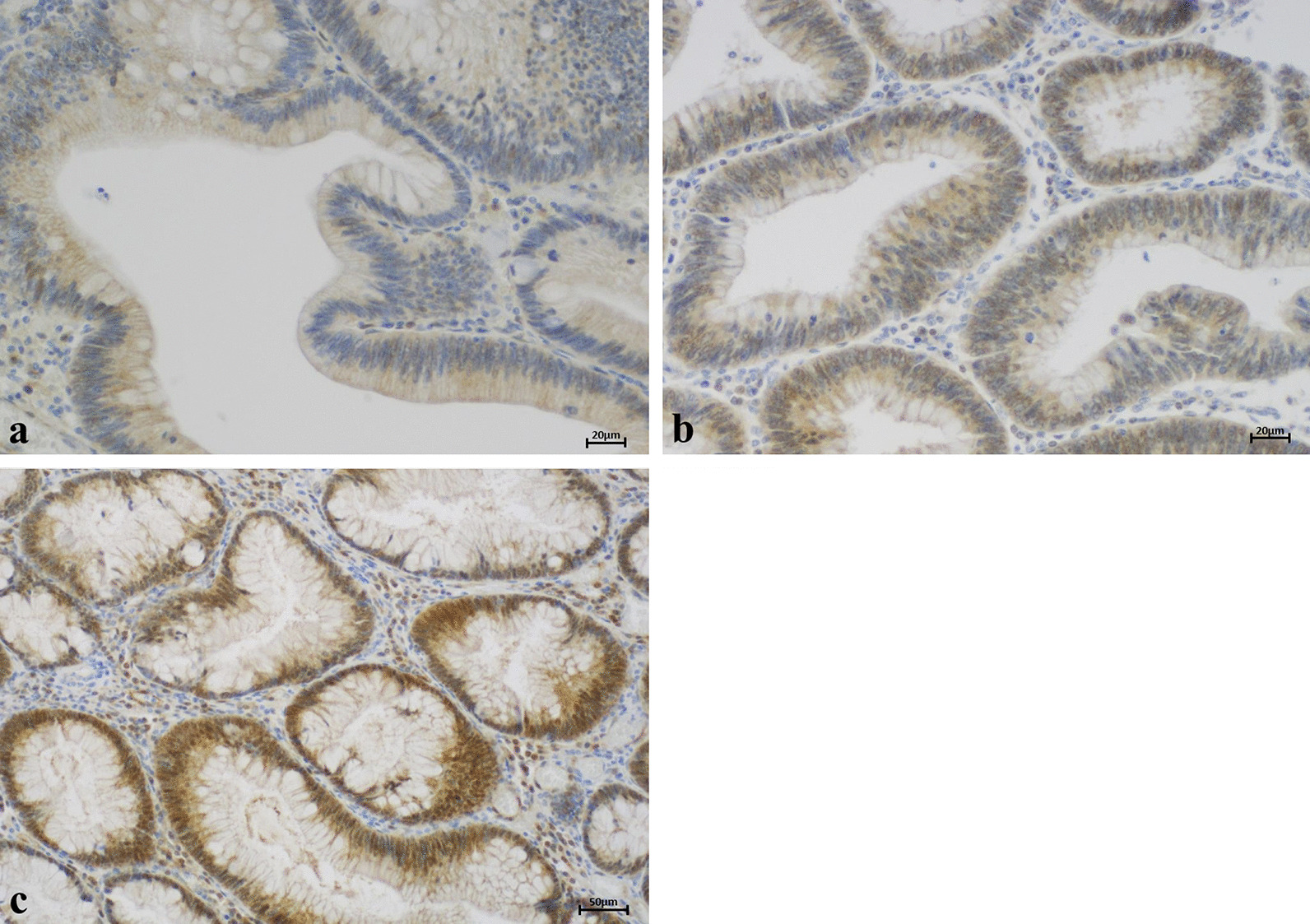
Representative examples of nuclear ARC staining. Most of the examined FAP adenomas showed overexpression of nuclear ARC. Only 6.6% exhibited no or very little nuclear ARC expression (score 0; **a**). In 62.7% of FAP adenomas, ARC was overexpressed in 10–75% of nuclei (score 1; **b**) and in 30.7% of adenomas, overexpression was present in over 75% of nuclei (score 2; **c**)

Of 217 FAP adenomas from 80 patients stained for ARC, 212 could be evaluated. The results of the ARC staining are shown in Table 1. Strong cytoplasmic ARC expression was detected in 32.5% (n = 69/212), and moderate cytoplasmic staining (score 2) in 49.1% (n = 104/212) of adenomas. In 18.4% (n = 39/212) of the adenomas, we found cytoplasmic staining equivalent to that in the non-tumorous mucosa (Fig. 1). Strong nuclear ARC expression (score 2) was found in 30.7% (n = 65/212) of adenomas. Moderate nuclear expression (score 1) was seen in 62.7% (n = 133/212), and no nuclear or weak ARC expression (score 0) was found in 6.6% (n = 14/212; Fig. 2). A highly significant correlation was found between the simultaneous expression of nuclear ARC and cytoplasmic ARC (*p* = 0.001; Table 2).

**Table 1:** Distribution of nuclear and cytoplasmic staining for ARC

ARC expression		ARC (cytoplasmic)				
		Score 0	Score 1	Score 2	Score 3	∑
ARC (nuclear)	Score 0	0	13	1	0	14 (6.6%)
	Score 1	0	23	65	45	133 (62.7%)
	Score 2	0	3	38	24	65 (30.7%)
	∑	0 (0%)	39 (18.4%)	104 (49.1%)	69 (32.5%)	212 (100%)

**Table 2:** Statistical correlation between nuclear and cytoplasmic ARC expression

		ARC (nuclear)
ARC (cytoplasmic)	Spearman correlation	0.277**
	Significance (two-sided)	0.001
	n	212

Expression of nuclear and cytoplasmic ARC was correlated highly significant (*p* < 0.01**)

### Expression of p53, Bcl-2, COX-2, nuclear β-Catenin and MMR proteins and their correlation with ARC

**Fig. 3:**
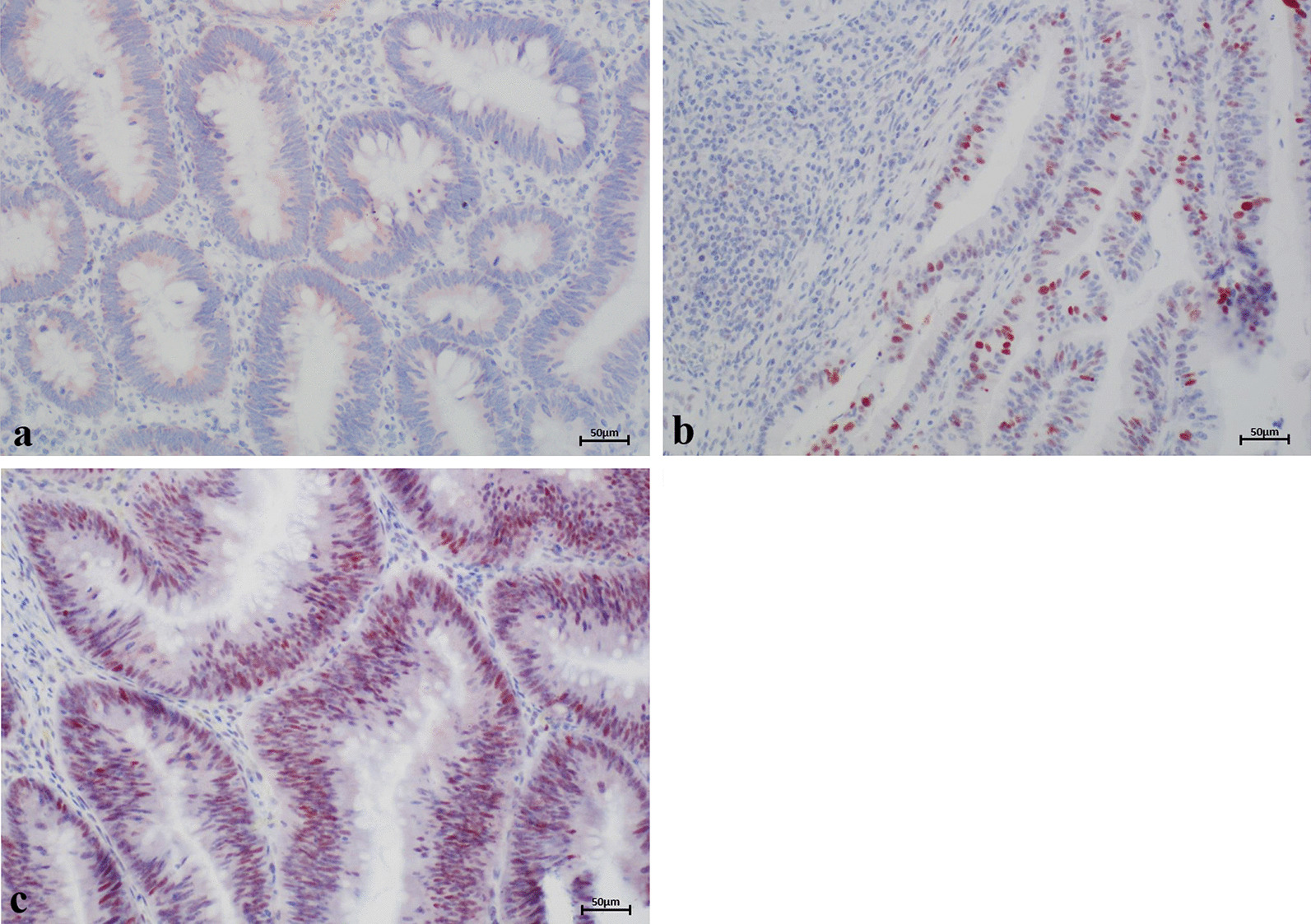
Representative examples of p53 results. Only 16% of FAP adenomas exhibited strong overexpression of p53 (score 2; **c**). However, overexpression of p53 correlated with nuclear ARC expression (*p* = 0.012). In most tissue slices, expression of p53 was limited to 10–75% of FAP adenoma cells (score 1; **b**). In 13%, no or weak p53 expression was detected (score 0; **a**)

Of 207 FAP adenomas analyzed, 27 (13.0%) were negative for p53, or expression was limited to less than 10% of adenoma cells (score 0). Score 1 and score 2 staining was observed in 147 (71.0%) and 33 (16%; Fig. 3) of adenomas, respectively (Table 3). P53 staining correlated with nuclear ARC staining (*p* = 0.012; Table 4) but not with cytoplasmic ARC staining.

**Table 3:** Evaluable immunohistochemical staining for p53, COX-2, Bcl-2 and nuclear β-catenin

	Score 0	Score 1	Score 2	∑
p53	27 (13.0%)	147 (71.0%)	33 (16.0%)	207 (100%)
COX-2	0 (0%)	44 (21.8%)	158 (78.2%)	202 (100%)
Bcl-2	8 (3.9%)	78 (38.4%)	117 (57.6%)	203 (100%)
Nuclear β-catenin	124 (59.0%)	62 (29.5%)	24 (11.4%)	210 (100%)

**Table 4:** Correlations between ARC and p53, COX-2, Bcl-2, nuclear β-catenin and MMR proteins (statistically significant results in bold type)

		ARC (cytoplasmic)	ARC (nuclear)
MLH1	Spearman correlation	− 0.028	0.074
	Significance (two-sided)	0.687	0.288
	n	207	207
MSH2	Spearman correlation	0.021	0.034
	Significance (two-sided)	0.763	0.624
	n	206	206
MSH6	Spearman correlation	− 0.004	0.071
	Significance (two-sided)	0.958	0.313
	n	205	205
P53 (score 2)	Spearman correlation	0.109	0.175^*^
	Significance (two-sided)	0.120	**0.012**
	n	204	**204**
Bcl-2	Spearman correlation	0.267^**^	0.142^*^
	Significance (two-sided)	**0.001**	**0.045**
	n	**200**	**200**
COX-2	Spearman correlation	0.066	0.149^*^
	Significance (two-sided)	0.357	**0.036**
	n	198	**198**
Nuclear β-catenin	Spearman correlation	0.019	0.249**
	Significance (two-sided)	0.780	**0.001**
	n	207	**207**

Nuclear ARC expression was found to correlate significantly (*p* < 0.05*) with the expression of Bcl-2, COX-2, with strong p53 expression and with nuclear β-catenin. In contrast, expression of cytoplasmic ARC only correlated with Bcl-2 but with none of other proteins; however, this correlation was highly significant (*p* < 0.01**)

**Fig. 4:**
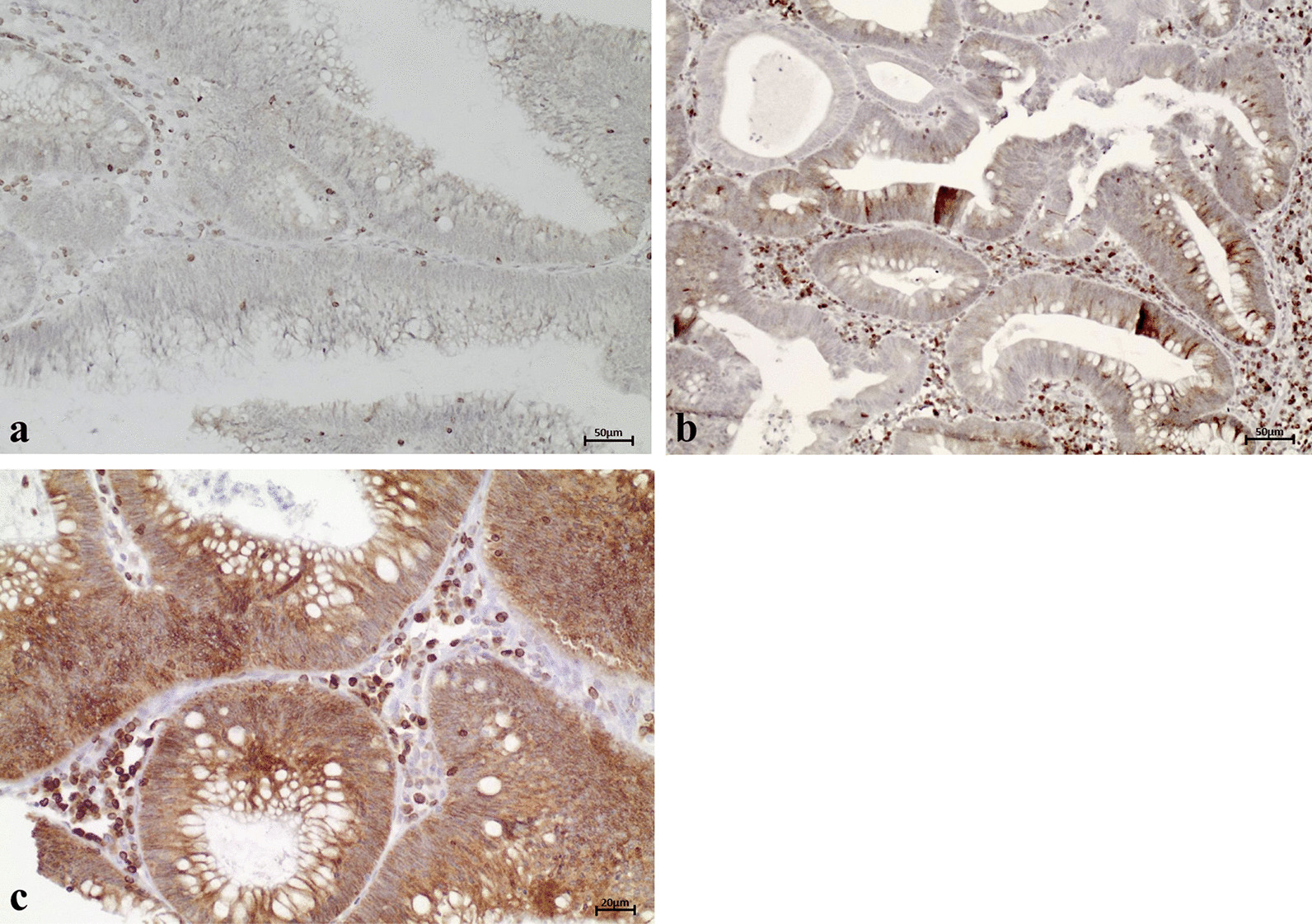
Representative examples of Bcl-2 results. In 3.9% of the adenomas, no Bcl-2 staining or staining in less than 10% of the tumor cells was observed (score 0; **a**). Bcl-2 was moderately overexpressed in 38.4% (score 1; **b**) and highly overexpressed in 57.6% of FAP adenomas (score 2; **c**)

We also tested whether ARC correlated with Bcl-2 expression in FAP adenomas. Score 1 and score 2 Bcl-2 staining was detected in 38.4% (n = 78/203) and 57.6% (n = 117/203) of adenomas, respectively. Score 0 staining (i.e., no staining or staining in less than 10% of tumor cells) was observed in 3.9% (n = 8/203; Fig. 4). A significant correlation was found between Bcl-2 staining and both cytoplasmic (*p* = 0.001) and nuclear (*p* = 0.045) ARC staining in FAP adenomas (Table 4).

**Fig. 5:**
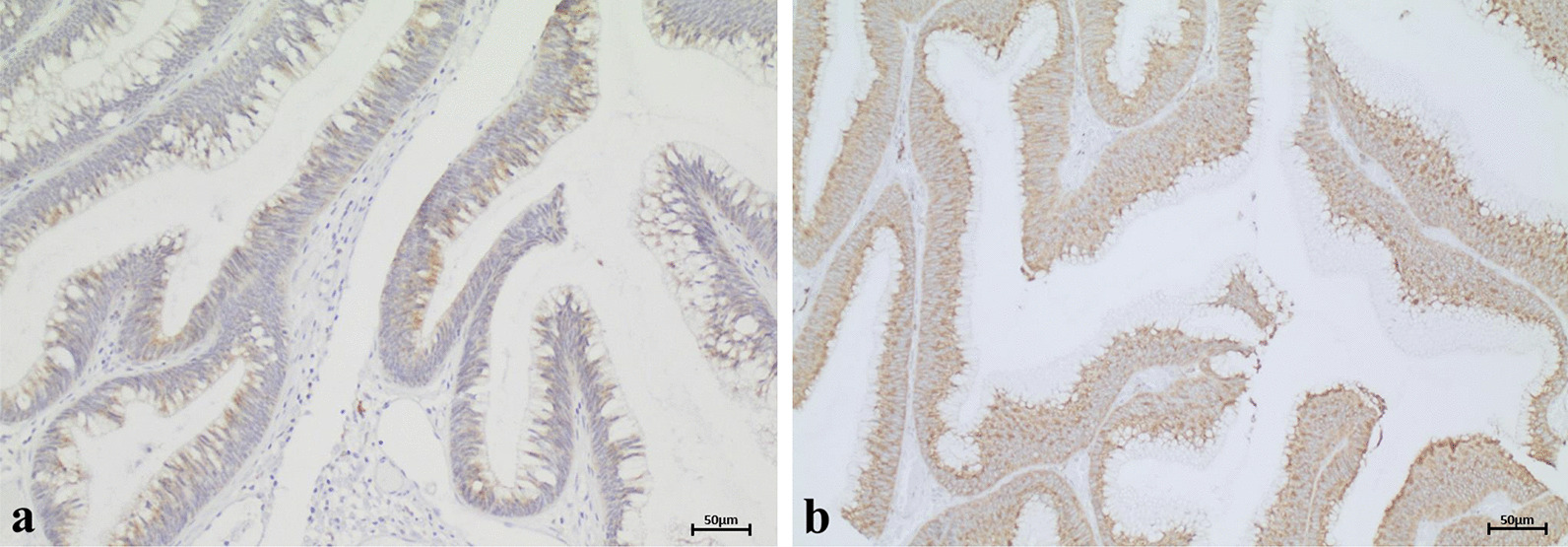
Representative examples of COX-2 results. All examined FAP adenomas exhibited COX-2 expression, which was moderate in 21.8% (score 1; **a**) of slices and strong in 78.2% (score 2; **b**)

For COX-2, score 1 and score 2 staining was detected in 21.8% (n = 44/202) and 78.2% (n = 158/202) of adenomas, respectively. Absence of staining or staining of less than 10% of tumor cells (i.e., score 0) was not observed (Fig. 5). Like Bcl-2, overexpression of COX-2 correlated with nuclear ARC expression (*p* = 0.036) but did not correlate with cytoplasmic ARC expression (Table 4)**.**

**Fig. 6:**
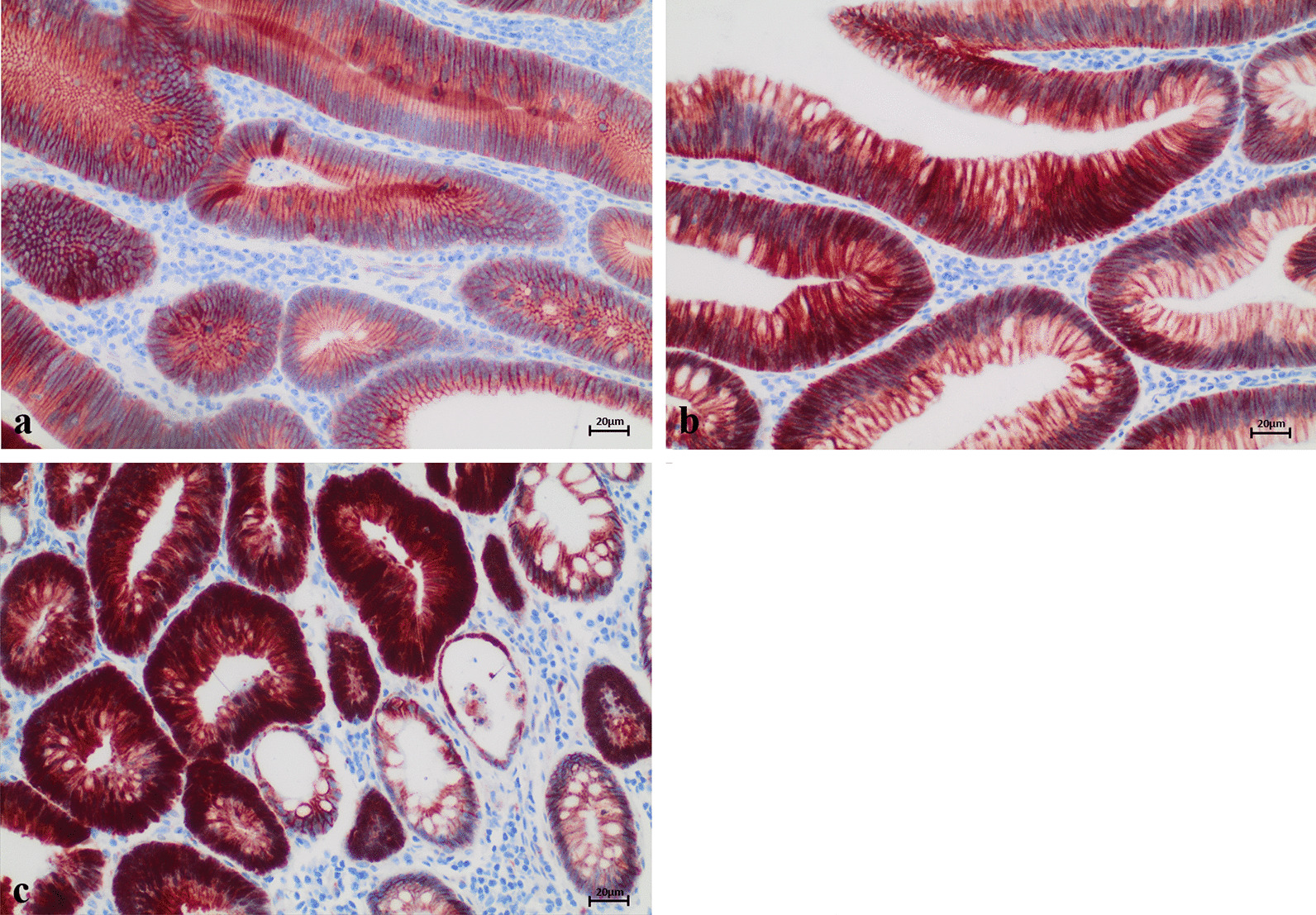
Representative examples of β-Catenin results. Cytoplasmic β-Catenin was accumulated in almost all FAP adenomas. In 59.0% of the adenomas, no nuclear β-Catenin staining or staining in less than 10% of the tumor nuclei was observed (score 0; **a**). Nuclear β-Catenin was moderately overexpressed in 29.5% (score 1; **b**) and highly overexpressed in 11.4% of FAP adenomas (score 2; **c**)

Cytoplasmic β-Catenin was accumulated in almost all FAP adenomas. For nuclear β-catenin, score 1 and score 2 staining was detected in 29.5% (n = 62/210) and 11.4% (n = 24/210) of adenomas, respectively. Score 0 staining (i.e., no staining or staining in less than 10% of tumor cells) was observed in 59.0% (n = 124/210; Fig. 6). Like Bcl-2 and COX-2, nuclear β-catenin expression correlated with the expression of nuclear ARC (*p* = 0.001) but did not correlate with cytoplasmic ARC expression (Table 4).

Neither nuclear nor cytoplasmic ARC correlated with any of the MMR proteins; however, this was due to the small number of MMR-deficient adenomas. Thus, an absence of staining for MMR proteins was only observed in 0.5–6.2% of slices (MLH1 6.2%, MSH2 0.5%, MSH6 2.9%). All but four patients were affected by sporadic MMR deficiency. Sporadic MMR deficiency could not be proved for these four patients because too few evaluable adenomas were available for them.

## Discussion

In this study, cytoplasmic overexpression of ARC was found in approximately 82% of FAP adenomas. This result is comparable to the findings of most previous studies on subcellular distribution of ARC and suggests that neoplasia resulting from germline mutations might not differ significantly from sporadic tumors with regard to ARC expression [11, 21–24].

In addition to cytoplasmic overexpression, nuclear ARC was slightly overexpressed in 62.7% and strongly overexpressed in 30.7% of FAP adenomas investigated in this study. These findings are in agreement with those of a previous study on ARC nuclear expression (21). However, reports on the intracellular distribution of ARC are controversial. For example, Wang et al. showed that ARC is almost exclusively located in the nucleus of both HeLa cells and human gastric cancer cells [25]. The observed differences regarding subcellular ARC location might be due to the use of cancer-derived cell lines. The role of nuclear ARC remains unclear, but intracellular ARC distribution can vary depending on the tumor type and whether human tumor tissue or tumor cell lines are evaluated. For example, a study on PC12 rat pheochromocytoma cells revealed a predominantly nuclear ARC location [41], whereas a different study on human melanoma cell lines detected a predominantly cytosolic and mitochondrial ARC location [42].

Nuclear ARC expression might also be a consequence of how ARC interacts with p53. ARC can bind to the tetramerization domain of p53 within the P/E region between amino acids 125 and 175; this triggers a nuclear export signal in p53 by disrupting p53 tetramerization [18, 29]. Consequently, higher intranuclear p53 expression levels might require more intranuclear ARC for inhibition and export. In our study, higher nuclear ARC levels were significantly (*p* = 0.012) more common in adenomas with high nuclear p53 overexpression, which are strongly enriched with *TP53* mutations. However, strong overexpression of p53 was detected in only 16.0% (n = 33/207) of all FAP adenomas in our cohort. This result is comparable to that of a previous study on p53 expression in FAP adenomas. It can therefore be suggested that p53 overexpression related to a mutation in *TP53* is uncommon in FAP adenomas and probably occurs later in the adenoma carcinoma sequence [36].

In our study, Bcl-2 was weakly overexpressed in 38.4% and strongly overexpressed in 57.6% of the FAP adenomas evaluated. These results are consistent with those from a previous analysis of various apoptosis markers in FAP adenomas [36]. Bcl-2 overexpression might substantially contribute to the development of FAP adenomas by means of its antiapoptotic potential. It has been shown to protect APC-deficient intestinal epithelial cells and intestinal stem cells from apoptosis and thus contribute to their malignant transformation [43]. In the current study, Bcl-2 overexpression correlated with the expression of both nuclear (*p* = 0.045) and cytoplasmic (*p* = 0.001) ARC. This might be a consequence of ARC-mediated nuclear hyperexport of p53, because less intranuclear p53 leads to reduced expression of the Bcl-2-neutralizing BH3-only proteins Bad and Puma [44]. Moreover, direct suppression of Bad and Puma is possible because ARC is able to bind both proteins via its CARD domain between amino acids 31 and 70, thus preventing their neutralizing effect on Bcl-2 [18].

Like Bcl-2, increased COX-2 expression has been observed in FAP adenomas before [30–33]. Likewise, in the present study, 78% of FAP adenomas exhibited strong COX-2 overexpression. Moreover, COX-2 overexpression correlated with the expression of nuclear ARC (*p* = 0.036). This correlation might reflect numerous connections both proteins were reported to be involved in. Previous studies on AML and mesenchymal stromal cells revealed that ARC is capable to induce the transcription factor NF-ƙB [45], which in turn induces the expression of COX-2 via transcriptional regulation of interleukin IL-1β [14]. ARC influence on COX-2 expression via NF-ƙB might also play a role in FAP adenomas, as both NF-ƙB and COX-2 are shown to be overexpressed in APC negative cases [46]. Conversely, it was shown that COX-2, also influences the expression of ARC via prostaglandin E2 (PGE2) [47, 48]. PGE2, which was also shown to be overexpressed in FAP adenomas [49], is capable to induce Ras and MAP-kinases (MAPK) in the Ras/Raf/MAPK pathway [47, 48] and β-Catenin in the Wnt/β-Catenin pathway [14]. As ARC was reported to be transcriptionally regulated by both pathways [13, 14], the correlation between COX-2 and ARC might be part of a feedback loop by which ARC levels may increase further. However, in FAP adenomas the Wnt/β-Catenin mediated regulation of ARC expression might be of particular relevance, as β-Catenin is accumulated due to its APC-mutation reasoned missing degradation [50, 51]. The effect of an aberrantly activated Wnt/β-Catenin pathway might therefore be enhanced by its PGE2-mediated increased induction in addition to the accumulation of β-Catenin. Regarding the subcellular localization of β-Catenin in FAP adenomas, however, there are different findings. Inomata et al. described nuclear expression in FAP adenomas and in resulting FAP carcinomas [50], while Kobayashi et al. detected nuclear accumulation only in FAP carcinomas but not in FAP adenomas [51]. We therefore also investigated, β-Catenin expression and localization in our collective. Furthermore, we checked for a possible correlation with ARC expression, as it was previously revealed in AML by Carter et al. Carter et al. didn´t differentiate between nuclear and cytoplasmic localization of ARC, thus, to the best of our knowledge, this is the first study, which investigated the correlation of β-Catenin and ARC with regard to the subcellular localization of ARC. In our study, β-Catenin was cytoplasmatic overexpressed in nearly all of FAP adenomas, which is in line with previous studies [50, 51]. Consequently, we focused on the nuclear expression of β-Catenin and found overexpression in 40.9% of FAP adenomas. The overexpression was moderate in 29.5% and strong in 11.4% of FAP adenomas. Discrepancies to the results obtained by Inomata et al. and Kobayashi et al. might be due to the differences in cohort size, which was much smaller in both studies compared to our study. We therefore concluded that nuclear β-Catenin accumulation is not only detectable in FAP carcinomas but already in FAP adenomas. Moreover, the highly significant correlation (*p* = 0.001) between nuclear β-Catenin and nuclear ARC suggested that ARC might be regulated by β-Catenin not only in AML-cells but also in FAP adenomas [14].

MMR deficiency was only detected in a few individual adenomas, which is consistent with the results from another study [52]. It can therefore be assumed that acquired MMR deficiency in FAP adenomas is very rare. Because the number of patients in this study with MMR deficiency was so small, it was not possible to conduct a more precise analysis of a correlation with nuclear and cytoplasmic ARC. However, it was noticeable that the average age of the patients with MMR deficiency (42.0 years) was higher than that of the patients without MMR deficiency (33.2 years). An age-dependent increase in the risk of MMR deficiency, as described for somatic cells [53], might therefore also occur independently of the existing FAP.

## Conclusions

The results of our study demonstrated both cytoplasmic and nuclear ARC overexpression in FAP adenomas. The highly significant correlation between nuclear ARC and nuclear β-Catenin also suggested that ARC might be transcriptionally regulated by β-Catenin in FAP adenomas. Because of its further positive correlations with p53, Bcl-2 and COX-2, nuclear ARC might play a substantial role not only in carcinomas but also in precursor lesions. However, further studies on the intracellular distribution of ARC are now required in order to investigate the different intracellular effects of ARC expression on precursor lesions and lesions with a higher grade of dysplasia.

## Data Availability

Not applicable.

## Abbreviations

AML: Acute myeloid leukemia
Apaf1: Apoptotic protease-activating factor 1
APC: Adenomatous polyposis coli
ARC: Apoptosis repressor with caspase recruitment domain
Bad: Bcl-2-Antagonist of Cell Death
Bak: Bcl-2 homologous antagonist/killer
Bax: Bcl-2-associated X protein
Bcl-2: B-cell lymphoma 2
CARD: Caspase recruitment domain
CK: Casein Kinase
COX-2: Cyclooxygenase-2
FADD: Fas-associated protein with death domain
FAP: Familial adenomatous polyposis
FFPE: Formalin fixed paraffin-embedded
MDM2: Mouse double minute 2 homolog
MLH1: MutL homolog 1
MMR: Mismatch-repair
MSH2: MutS protein homolog 2
MSH6: MutS protein homolog 6
Puma: P53 upregulated modulator of apoptosis
Ras: Rat sarcoma
Raf: Rat fibrosarcoma
RTU: Ready to use
TMA: Tissue microarray
Wtn: Wingless-Protein
